# Do biodiversity monitoring citizen science surveys meet the core principles of open science practices?

**DOI:** 10.1007/s10661-022-10887-y

**Published:** 2023-01-12

**Authors:** Samantha Suter, Brian Barrett, Natalie Welden

**Affiliations:** 1 School of Interdisciplinary Studies, University of Glasgow, Glasgow, UK; 2 School of Geographical and Earth Sciences, University of Glasgow, Glasgow, UK

**Keywords:** Citizen science, Open science, Environmental monitoring, Public, Volunteer, Data

## Abstract

Citizen science (CS), as an enabler of open science (OS) practices, is a low-cost and accessible method for data collection in biodiversity monitoring, which can empower and educate the public both on scientific research priorities and on environmental change. Where OS increases research transparency and scientific democratisation; if properly implemented, CS should do the same. Here, we present the findings of a systematic review exploring “openness” of CS in biodiversity monitoring. CS projects were scored between − 1 (closed) and 1 (open) on their adherence to defined OS principles: accessible data, code, software, publication, data management plans, and preregistrations. Openness scores per principle were compared to see where OS is more frequently utilised across the research process. The relationship between interest in CS and openness within the practice was also tested. Overall, CS projects had an average open score of 0.14. There was a significant difference in open scores between OS principles (*p* =  < 0.0001), where “open data” was the most adhered to practice compared to the lowest scores found in relation to preregistrations. The apparent level of interest in CS was not shown to correspond to a significant increase in openness within CS (*p* = 0.8464). These results reveal CS is not generally “open” despite being an OS approach, with implications for how the public can interact with the research that they play an active role in contributing to. The development of systematic recommendations on where and how OS can be implemented across the research process in citizen science projects is encouraged.

**Supplementary Information:**

The online version contains supplementary material available at 10.1007/s10661-022-10887-y.

## Introduction

### Open science and research democratisation

The increasing efforts to democratise science and its outputs have resulted in the rapid development of novel research practices and resources (Mirowski, 2018; Strasser & Haklay, 2018). One coordinated approach by which we may improve the accessibility and transparency of research to readers of all backgrounds is the open science movement. Open science (OS), although not a new term, may be hard to categorise under a single definition. However, it can be generally understood in its aim to increase the availability of all scientific research to general society (be that policymakers, laymen, or other researchers) or to develop a “transparent and accessible knowledge that is shared and developed through collaborative networks” (Vicente-Sáez & Martínez-Fuentes, 2018). Proponents of OS argue that this availability leads to increased reproducibility of research as the inquiring body has access to the entire research process, including data, code, methods, analysis, and results (Taylor et al., 2017).

The OS movement has become ever more important with increased public interaction with research, both through social media and through continuous news in the online era. Research affects society via its potential to inform policy, influence the economy, design technology, and effect sociality. Research is frequently funded by public investment from the taxpayer; for example, in 2017, £9 billion was spent on research and development in the UK (The Royal Society, 2019). The UK government also increased this spending by 15% in 2021, meaning the return of investment should be even greater for the public (Stokstad, 2020). However, there are numerous barriers between scientific research and the successful implementation of research findings (e.g. in policy) which may be addressed using OS practices. This has previously been demonstrated in the development of conservation policies using open data collected by the citizen science project “eBird”, turning the research into tangible achievements (Sullivan et al., 2017). Practicing OS has also increased wide collaboration, as seen in the development of the Zika, Ebola, and COVID-19 vaccines, providing novel mRNA methods which now shape the future of disease response (Burgelman et al., 2019; Edwin et al., 2020; Pardi et al., 2018).

Research’s reliance on public funding and potential societal impact underlines the importance for every individual, no matter their role in society, to have access to the research that shapes their lives. Nevertheless, research is seldom available to those it influences. Indeed, access to both publications and data remain stubbornly uneven across the scientific community, and research not available to the whole scientific community is also less likely to be available for the public (Scaria & Rangarajan, 2016). However, the OS movement is not only economically appealing, due to the apparent return on investment that research may bring (e.g. in the creation of new products or industrial innovation), it also fosters trust, enhanced knowledge, and awareness of what the public are contributing towards (Grand et al., 2012; Houghton et al., 2010; OECD, 2015).

### Open science and research integrity

Application of OS also addresses barriers present when seeking to determine the reliability of research. For example, lack of reproducibility in research prevents work from being verified, replicated, and expanded upon (Scaria & Rangarajan, 2016). OS can improve the quality and reliability of research through increased opportunity for peer review, method replication, and collaboration. As this can increase the robustness of the methodology, published reports may be at reduced likelihood of being retracted. Additionally, papers which had associated open data were even less likely to be retracted (Lesk et al., 2019). This has also been found with research that linked preprints, with 0.03% retracted compared to studies without preprints with a retraction rate between 0.04 and 0.06% (Avissar-Whiting, 2022). Where retractions are due to scientific misconduct, increased transparency in research should improve research integrity and reduce retractions overall (Marcus & Oransky, 2014). In fact, Marcus and Oransky (2012) called for journals to have a transparency index, essentially a metric that measures processes in journal publishing, including the employment of OS practices. In addition to increasing the reliability of the publication base, the practice of OS has additional benefits to researchers: increasing the publication of null findings, increasing citations from preregistrations, improving researcher rights, and heightening collaboration leading to greater research efficiency (Clements, 2017; Franco et al., 2014; Hajjem et al., 2006; Levin et al., 2016; McKiernan et al., 2016).

### Open science approaches

The practice of OS may be divided into five “advocacy schools”, including (a) public influence on, and understanding of, scientific research; (b) the accessibility to both re-use raw data and retrieve published results; (c) the architecture surrounding the storing and dissemination of research, (d) the collaboration between different parties to increase both inputs and outputs of the research, and (e) how to measure the research impact (Fecher & Friesike, 2014). Although, implementation of the aims of these OS schools will be specific to the area of the research process in question.

Various models have been proposed to highlight OS approaches (Table 1). The relative newness and broadness of OS, however, has meant that there are no strict guidelines to follow for practising OS. Therefore, the application across the multiple models is often quite specific. For example, Klein et al. (2018) produced a framework for OS in psychological research to demonstrate how, and where, to open the research process, whereas Ayris et al. (2018) defined 8 pillars of OS for university practice. Ayris et al.’s (2018) guidelines are less specific in their instructions on where OS can be implemented but instead broadly examine what it means to be practising within OS. Other models, such as Bowman and Keene’s (2018) “onion” model and de la Fuente’s (2019) “beehive” model do highlight specific practices, however, are less obvious in where these fall within the research process. Common OS practices do appear across the models which can be used to create a general framework to highlight where OS practices can be used across the research process.

**Table 1:** Published open science practices and their applications in research

OS practice	Application in source	Source
FAIR Data, Research Integrity, Next Generation Metrics, Future of Scholarly Communication, Citizen Science, Education and Skills, Rewards and Initiatives, and EOSC	University practice, research methodology	Ayris et al., 2018
By author request, shared materials, shared analysis, shared data, preregistered reports	Cross-disciplinary, research methodology	Bowman and Keene, 2018
Open notebooks, open data, open peer review, open access, open source, scientific social networks, citizen science, and open educational resources	Cross-disciplinary	de la Fuente, 2019
Data management plan, published preregisters, materials (data and scripts) on public repository,	Psychology research process	Klein et al., 2018

OS practices at the start of the research process include the formulation of data management plans (DMP) and preregistrations. DMPs are of increasing importance to many journals and funding bodies and allow the researcher to consider how they will handle, store, and share the data collected during a study. Creating a DMP allows OS to be considered at the start of a project and ensure that it can be practiced throughout the research process (Williams et al., 2017). The process of locating DMPs must be straightforward to improve efficiency and, as such, the adoption of a key sharing platform should be recognised. In the UK, many institutions encourage the use of DMPonline as a major sharing platform (Simms & Jones, 2017). Preregisters require detailing on hypotheses to be tested, methods for data collection, and the analysis to be undertaken. By describing how the data is going to be analysed, it removes the possibility of changing the analysis method depending on the study findings or generating hypotheses after the results were found (known as HARKing) (Parker et al., 2019). Preregisters use online platforms for sharing, for example, the largest being the Open Science Framework (Kupferschmidt, 2018).

Discoverable research outputs are the most widely practiced aspects of OS. As indicated above, open data enables research to be replicated and verified, as well as increasing collaboration. There are many platforms available to store and publish data (such as, DataONE and the Register of Research Data Repositories) where data can be located across several repositories (Michener, 2015). Similar tools can be used to publish methods and code for data analysis but it is important to make sure that the analytical programmes used is accessible to all, for example, with the use of open-source data analysis software. However, the sensitivity of data must be considered and may form a constraint on what data can be shared. Recognition of which has given rise to the term “as open as possible and as closed as necessary” (European Commission, 2016). Finally, there is the focus on open access results. Many journals have subscription fees or one-off payments to allow access to a published paper. This reduces potential engagement with other researchers and the public (Peterson et al., 2019). Access to publications is the most frequently prioritised OS practice; however, it is important to have all OS tools in place throughout the research process; otherwise, each area will lose significance. The entire research process and the outputs must be transparent to reproduce, verify, and expand upon research.

### Citizen science as an open science practice

Science communication and public participation are both emphasised in the practice of OS. Citizen science (CS) involves the public in the scientific research process, most commonly for data collection and analysis purposes (Cohn, 2008), thus integrating both of these elements. The collaborative aspect of CS may overcome the disconnect between the scientist, the policymaker, and the public through collaboration during the research process (Cavalier & Kennedy, 2016). In this manner, CS both enables public access to research and integrates knowledge exchange, allowing contributions from the public (Hecker et al., 2018). This betters the efforts of OS at growing science democratisation through increased knowledge exchange, understanding of the scientific process, and diverse representation, with research that is more aligned with the public interest (Strasser & Haklay, 2018). The greatest benefit of CS in OS may be seen in the public advocacy school. For example, for species monitoring in Europe, there are 18 citizen scientists for every 1 research scientist (Groom et al., 2017). It is important to harness this enthusiasm from the public to generate idea creation and democratic governance (Storksdieck et al., 2016).

An important contribution of CS to OS practices should be the increased availability of the data to potential collaborators who are not involved directly with the research. Many of the largest CS projects are in biodiversity monitoring (Kullenberg & Kasperowski, 2016). Indeed, such collaboration is essential in tackling the current biodiversity crisis (Costello et al., 2015). Both CS, and more broadly OS, enable new partnerships to address the gaps in global biodiversity monitoring. Firstly, OS can do this by developing platforms, such as the Group on Earth Observations Biodiversity Observation Network or Collect Earth, where common data and information is shared (Pettorelli et al., 2014). Secondly, CS can then combine datasets across a larger scale, for example, where the Euro Bird Portal in Europe and the Second Southern African Bird Atlas Project in Africa pooled data across multiple countries to compile one large dataset (Amano et al., 2016).

As indicated above, OS principles require that research should be reproducible and that data be accessible, and it may be assumed that CS adheres to these principles. However, Groom et al. (2017) found that CS data scored the second lowest on an open data index concerning biodiversity observations. Reasons for this included licencing restrictions, surrounding landowner permissions, concerns from data holders on data sharing, and funding disincentives (Groom et al., 2017). However, open CS data has the potential to provide large amounts of data which may otherwise not be achieved, this was seen in a water monitoring scheme in the US, where the CS data was made open, forming more than 50% of observations (Poisson et al., 2020). If CS data remains closed, this can restrict further research on a topic by hindering collaboration. To overcome this issue, the reasons for reduced data sharing need to be addressed. For example, how can sensitive data be protected if it is openly available? Once these issues have been addressed the benefits of OS can be harnessed.

### Aims and objectives

For CS to fulfil its potential as a core practice in OS, CS projects must adhere to the full range of OS principles. Although previous studies have indicated the inaccessibility of CS data, CS projects have not been assessed in all areas of the research process. As such, this study aims to systematically review biodiversity monitoring CS projects to determine whether they meet the core principles of OS. The specific objectives are to:

i) Rate environmental CS projects on their “openness” across the entire research process
ii) Determine how open each aspect of the research process is across CS projects
iii) Investigate if CS project “openness” has increased with a rise in citizen science

## Methods

### Selection of studies

A systematic review was undertaken to identify publications arising from CS projects on biodiversity. The key words “citizen science” and “biodiversity monitoring” were used to search Web of Science. This database was chosen due to its common use in systematic reviews, specifically surrounding biodiversity conservation research, and the large number of results generated in the initial search (Boice, 2019). Results were initially filtered to include only journal article submissions before 2022.

**Fig. 1:**
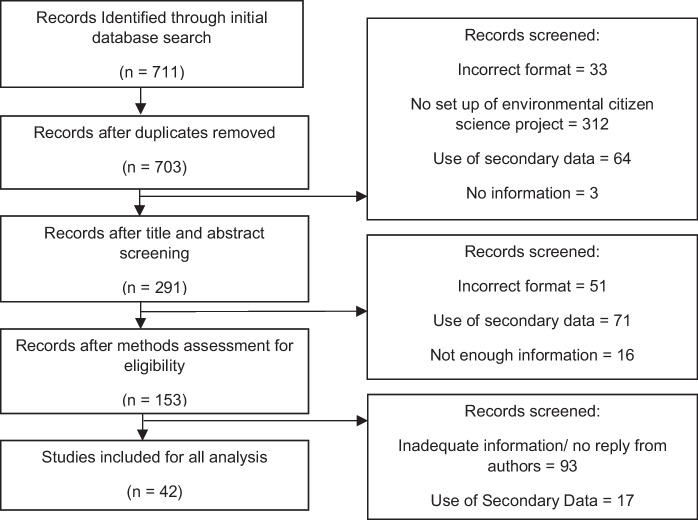
PRISMA diagram of the selection and exclusion process of included papers in the systematic review

Subsequently, the results were exported to EndNote and duplicates were removed, before being sorted by title and abstract to exclude review papers, book entries, irrelevant studies (e.g. studies which did not consider CS or biodiversity monitoring), studies using secondary data or data not based on the authors’ own CS projects, or studies that were based on development of certain aspects of a CS project (for example, an app). Where it was unclear whether these criteria were met, the publication was carried over into the subsequent sorting stage. The methods sections of each paper were then reviewed, and those papers which did not have enough relevant information to meet the criteria above were excluded. The number of papers returned at each stage of this sorting process can be seen in Fig. 1.

Due to a large level of incomplete information (especially regarding data management plans and preregisters), the authors of the 153 papers identified from the methods sorting were all contacted regarding missing information. Authors were given a month to respond to the emails before being excluded. Projects by authors that gave complete answers were included for analysis (Fig. 1).

### Open science criteria

The CS projects identified were assessed based on OS criteria. These included whether studies had associated DMPs, preregisters, available data and code, free software for analysis, and open findings available to everyone. Projects were assessed to see how many of the open science principles were met out of the six. Projects were then scored based on how open each principle was; closed was given a score of − 1, partially open was given a score of 0.5, and fully open was given a score of 1 (Table 2). Where the OS principle was not applicable (for example, where projects used software that does not require code, or where simple descriptive statistics were used which did not require specific packages), these were left blank.

**Table 2:** Open science criteria used to rate the openness of biodiversity monitoring Citizen Science projects between − 1 and 1. Each criterion was scored from − 1 (closed), 0.5 (partially open), and 1 (open). Where information was not applicable to the project, these were left blank

**Open science criteria**	**Closed (***− ***1)**	**Partially Open (0.5)**	**Open (1)**
Data management plan	None	Accessible by request/internal	Discoverable (e.g. DMPonline/DMPtool)/attached
Preregistration	None	Accessible by request/internal	Discoverable (e.g. Open Science Framework)/attached
Open data	None	Accessible by request/internal	Attached/online repository
Open code	None	Accessible by request/internal	Attached/online repository
Open software	Subscription software	Transferable	Free software
Open access	Behind paywalls/subscriptions	Partial Access	Open access journals/websites for results

### Data analysis

All statistical analysis was conducted in RStudio (v3.6.3). To rate CS projects on their “openness” across the entire research process, the scores for each project (*n* = 42) were averaged to give a value between − 1 to 1, with higher values signifying more open projects. Mean score was selected rather than total score as not all categories were relevant to each paper (for example, open code), and the use of totals artificially penalised these studies. Assumptions for normality could not be met, and a non-parametric Kruskal Wallis test was used to evaluate whether any of the OS principles were more commonly applied. A linear regression analysis was used to investigate if CS project “openness” has increased as the number of CS project has risen. All significance levels were set to 0.05.

## Results

Analysis was conducted on 42 biodiversity CS projects published between 2005 and 2021 (see supplementary material), with an average openness score of 0.14 (minimum − 0.67, maximum 0.9) across all projects for all OS principles combined. However, the number of papers (*n* = 153) identified during the review process was initially much higher, with many papers discounted due to missing information. For example, only 36.6% of the original 153 papers had a data availability statement and/or a supplementary material section. However, this did not always detail whether either the full data and/or code or preregisters and/or data management plans were attached or completed. The authors of these papers were contacted in the event that they should wish to provide further detail regarding their studies. All original 153 authors were contacted; 69 responded; however, only 42 projects provided complete information. The only OS practice that could be scored without correspondence was the openness of findings (i.e. was the paper published under an open access license or not). Of the original 153 papers, 152 papers had to be contacted regarding preregisters.

### Adherence to open science principles

**Fig. 2:**
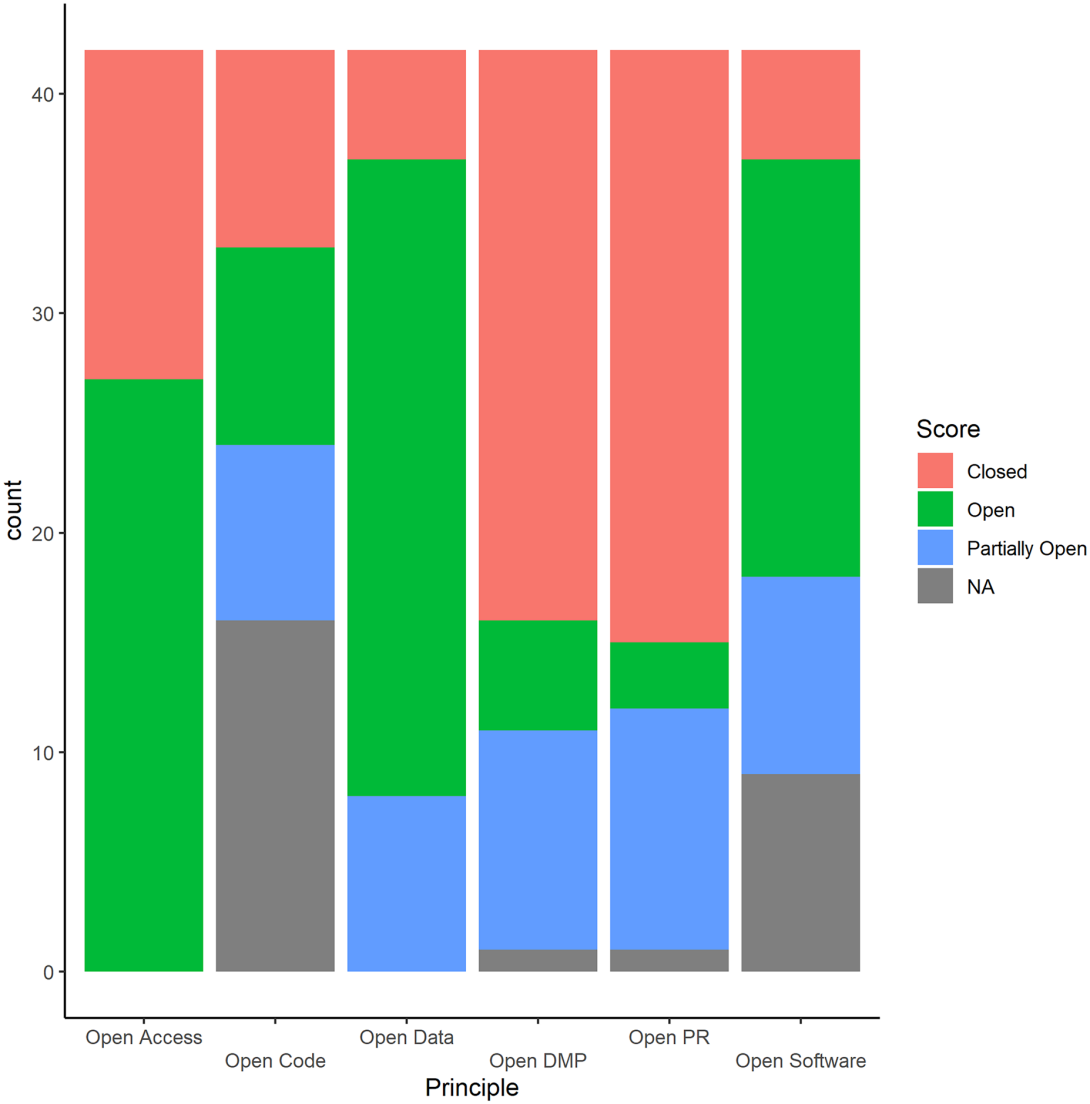
Distribution of openness scores across biodiversity monitoring citizen science projects per open science principle

There was a large variation in adherence to OS principles across projects (Table 3). The distribution of openness scores across projects, per OS principle can be seen in Fig. 2. The most regularly adhered to OS principle was open data (69%), followed by open access where 64% of papers had open access publishing. The percentage of projects that had fully open software was 58%, whereas 35% of projects had fully open code, 12% had fully open DMPs, and just 7% had fully open preregisters.

**Table 3:** The number of citizen science projects distributed by their openness across open science principles

		**Number of projects by openness score**		
**Open science principle**	**Number of projects per principle (after NA removal)**	** − 1 (Closed)**	**0.5 (Partially Open)**	**1 (Open)**
Open access	42	15 (35.7%)	0 (0%)	27 (64.2%)
Open code	26	9 (34.6%)	8 (30.8%)	9 (34.6%)
Open data	42	5 (11.9%)	8 (19%)	29 (69%)
Open DMP	41	26 (63.4%)	10 (24.4%)	5 (12.2%)
Open preregistration	41	27 (65.9%)	11 (26.8%)	3 (7.3%)
Open software	33	5 (15.2%)	9 (27.3%)	19 (57.6%)

### Average openness

Mean openness scores for each principle across the 42 projects were calculated after removal of non-applicable categories. The OS principle which is most frequently employed is the use of open data with a mean score of 0.67 (± 0.65 SD) across projects. The open science principle that is least employed is the use of preregisters with a mean score of − 0.45 (± 0.78 SD) across projects (Fig. 2).

The results of the Kruskal–Wallis test showed that the difference in openness scores between OS principles was significant (*p* =  < 0.0001, *df* = 5, *F* = 60.002). A post hoc Dunn’s test with Bonferroni correction showed there was a significant difference in scores between open DMP and open access principles (*p* = 0.0006), open preregistrations and open access principles (*p* = 0.0001), open DMP and open data principles (*p* =  < 0.0001), open preregistrations and open data (*p* =  < 0.0001), open software and open DMP principles (*p* = 0.0001), and open software and open preregistration principles (*p* =  < 0.0001).

### Open science within citizen science

The number of CS projects covered in this review increased from 1 project in 2005 to 11 projects in 2021. Over this period, the average openness score appeared to decrease, although this was not seen to be significant (*p* = 0.9706, *DF* = 40, *F* = 0.00138). We also investigated whether openness in CS was influenced by the apparent interest in the field. The results show there is not a significant correlation between project openness per year and the number of CS projects per year (*p* =  < 0.8464, *DF* = 9, *F* = 0.03974) (Table 4). No one OS principle appeared to have a greater influence on this result, as none of the principles present a trend in use with the rise in CS projects. This was further determined by a linear mixed effect model, where principle was included as a random effect and accounted for 22% of the variance within the model. As such, it was removed from the final linear regression.

**Table 4:** Open science principle yearly average open score by the number of citizen science projects per year between 2005 and 2021

		Open science principles					
Year	Number of projects	Open access	Open code	Open data	Open DMP	Open PR	Open software
2005	1	− 1	NA	1	0.5	1	1
2010	1	1	1	1	− 1	− 1	NA
2011	1	− 1	− 1	− 1	− 1	− 1	1
2012	1	− 1	0.5	1	− 1	0.5	− 1
2015	3	− 0.33	− 1	0.33	− 0.33	− 1	0
2016	4	1	0.5	0.75	0.38	0.17	0.5
2017	6	0.67	0	0.75	− 0.25	− 0.5	0.38
2018	2	1	0.75	0.75	0.5	− 1	1
2019	4	0	0.86	1	− 0.5	− 0.13	0.88
2020	8	0.25	0.36	0.94	− 0.38	− 0.44	0.58
2021	11	0.27	− 0.19	0.41	− 0.82	− 0.59	0.6

## Discussion

### Implications of results

The application of OS principles in CS projects have not been widely investigated beyond the scope of data assessments (Borda et al., 2020; Williams et al., 2018). This makes comparisons among research difficult and suggests why this review is important in identifying where best practice methods are underutilised. OS practices are difficult to outline due to the varying nature of research questions and disciplines, suggesting that what is applicable to one research question may not be suitable for another. Subsequently, researchers may have different opinions on what is considered an OS practice because of the lack of consistent guidance.

The results presented above indicate a lack of consistency in the number and extent to which OS principles are commonly implemented in CS research. Open access and open data were far more common in the research process compared to other practices, with these principles also having the highest mean openness scores found in this review. Bowser et al. (2020) found even higher levels of discoverable data where 75% of 36 projects (across a range of scientific disciplines) made the CS raw data available by some means. However, the remaining lack of access to information (publications and data) was still noted as one of the bigger downfalls in CS projects in comparison to others, for example, data quality measures. This is supported by Groom et al. (2017) who found that CS data scored the second lowest on an open data index concerning biodiversity observations. OS practices (specifically around open access and open data) in biodiversity conservation have long been called for (Fonseca & Benson, 2003; Gaikwad & Chavan, 2006; Mose et al., 2018). However, although open data and open access practices may score higher than other assessment criteria in reviews, they are by no means practiced on a large scale. Preregistrations and DMPs were used the least frequently. Very few studies have investigated the use of these within CS projects. One report based on CS projects across disciplines found that DMPs were implemented in 60% of projects (although it is unclear if these were publicly available) and 38% had raw open data (Schade & Tsinaraki, 2016).

Although the findings in the other studies outlined above are not directly comparable to our results due to differences in study identification methods and analysis, together they do show there are still large gaps in the utilisation of OS principles. The drivers for these findings appear largely to be what is common versus what is not. Through correspondence with authors, it was noted that many researchers were unaware of certain OS practices, predominantly preregisters, making it impossible for them to be utilised. This may be the result of the lack of common practices, education, or guidelines surrounding the OS process. Similar issues were observed in relation to DMPs. DMPs can be made readily accessible by platforms such as on DMPOnline, and preregisters can be published on the Open Science Framework, but less than 1% of papers in this review utilised these resources. Lack of guidance across scientific organisations, journals, or funders can result in fewer incentives or reduce researcher ability to contribute to OS. Allen and Mehler (2019) implied that this is likely to improve with time and greater interest and investment in OS but this has not been assessed. Therefore, it can be suggested that the absence of an OS practice may not be an active choice researchers make but simply lack of awareness surrounding the movement.

Another common theme that appeared to limit the implementation of OS practices was the lack of funding (pers. Comms). Unfortunately, OS practices often come with associated costs, for example, that of open access publishing. Many researchers are deterred by this if they do not have the resources (particularly seen in the Global South) meaning that participation in OS is not feasible or possible for all (Fontúrbel & Vizentin-Bugoni, 2021; Nabyonga-Orem et al., 2020). Conversely, access to funding, or lack thereof, could explain the relatively high proportion of studies which utilised open software, identified as the third most adhered to OS principle, as open software benefits the researcher in that it is free for them to use. This review discovered that where open data or code was used, these were found in public repositories such as GBIF, iNaturalist, github, the project’s website (if applicable), or included in supplementary materials. Open software largely included R and QGIS. However, although there is software available for OS (e.g. storage, validation, and dissemination sites), many are still in the process of being created or are comparatively unknown.

It must also be noted that increased research openness may lead to increased competition. OS processes are sometimes considered more time-consuming due to the preregistration and documentation procedures involved, creating concern around reduced publications or the prospect of being “scooped” by other authors (Allen & Mehler, 2019). However, this may be counteracted by the possibility of increased citations from the open-source documents and time reduction from collaboration opportunities. Additionally, if copyright laws that are associated with open access publishing are applied to all stages of data sharing, then the possibility of a researcher’s work being taken is minimised (Levin et al., 2016).

Funding disincentives, time commitments, and lack of awareness imply that the widespread implementation of OS remains a low priority in biodiversity research, which is supported by this review. Previous research has indicated a rise in CS since the twentieth century, with the largest application in ecological monitoring: attributed to many reasons including the low-cost, efficient methods it provides, or increased interest from the public (Kelemen-Finan et al*.,* 2018; Kullenberg & Kasperowski, 2016; Toerpe, 2013). However, the increased interest in CS has not translated to a greater adherence to OS principles within the field. Potentially, this could also be related to a lack of want within the scientific field where OS is not an accepted practice. Although this result cannot be compared to other studies, it is not what was initially expected.

In this era of “big data”, it was hypothesised that project openness may have increased in practice, as CS itself can facilitate OS (Bezjak et al., 2018). Nevertheless, a number of meaningful restrictions on data availability remain, for example, both ethical and GDPR concerns where working with human participants makes the ethics around sharing data difficult (Suman & Pierce, 2018). As the projects are biodiversity based, there are concerns regarding the potential unintended secondary effects of data sharing, e.g. highlighting locations of rare and endangered species (Ganzevoort et al., 2017). This does not mean that all principles of OS should be disregarded and justifications for certain closedness in projects should be made apparent. The results here support the notion that scientific research is often based on quantity not quality, where the number of publications and citations is seen as a measure of success (Fire & Guestrin, 2019). This is further evident from the findings that even retracted papers are still heavily cited and not removed if cited before a retraction occurs (Bolland et al., 2022). One reason for this is that the retraction status of a paper is usually unknown to authors (Teixeira da Silva & Bornemann-Cimenti, 2017). Methods have been proposed where journals use software that detects plagiarism (already utilised by some) and pairs this with retraction databases, such as Retraction Watch, to increase retraction clarity (Cosentino & Veríssimo, 2016). As such, OS has the potential to increase the transparency of retractions and heighten scientific credibility overall. This is especially the case when meeting the expectations of OS guidelines, i.e. not take advantage of open practices such as in the publication of preprints without any peer review, which was seen during the COVID-19 pandemic (Rzymski et al., 2020). It is with hopes that OS can reduce the impact of “infodemics” and reduce scientific misinformation, not contribute to it (Pool et al., 2021).

### Limitations

This review focused on patterns of openness in biodiversity monitoring CS projects. While over 150 papers were identified in our literature review, not all the eligible studies could be included in our analysis due to incomplete project information/no response from authors. Additionally, due to time considerations, the influence of project structure on the resulting project openness and the factors which enable or are otherwise responsible for successful incorporation of OS principles were not explored. Future research may focus on these areas to target suggestions more specifically. It must also be noted that the purpose of this review is not to criticise the assessed projects for their adherence, or lack of, to OS practices, but to highlight areas for improvements that may be made in the field at large.

The scope of the included projects was wide; some projects aimed to create a CS project to assess this method as a valid tool for biodiversity monitoring, and other projects used CS projects as a secondary goal, primarily focusing on analysis of the ecological data collected. It would be reasonable to assume where the development of a CS tool for biodiversity monitoring was not a primary goal of the research, OS itself would not have been a main consideration. Included papers also comprised a large geographic area with projects based on every continent. There is the possibility that the practice of OS does not translate globally, especially with a lack of common guidelines, language barriers, and issues regarding access to OS tools (technological challenges) and funding.

Project duration also varied from one day biodiversity counts to multi-decade and ongoing projects. It was not in the scope of this systematic review to determine whether project duration influenced adherence to OS practices. There is potential for projects (particularly ongoing ones) to move towards open science approaches. Even completed projects may still make previous data and code open, for example. However, this relies on a greater change in mindset and acceptance of new research practice as beneficial for scientific research and public engagement in science.

### The future of open science

The successful application of OS principles is dependent on the existence and visibility of appropriate tools in combination with standard guidelines. These necessary tools for OS are mainly in the form of online repositories and databases to store data, social media platforms for sharing data, and free access journals to present research outcomes (Neylon & Wu, 2009). Although such tools are now more readily available, there are still barriers which arise because of different policies across journals, funders, and governing parties. However, due to the vast expanse that scientific research covers and the variety within scientific processes, flexibility is required when applying practices across disciplines. Universal OS practices may not apply to all stages of research and data management, requiring different tools. It may be more advantageous to actively encourage OS through policies, institutions, and funding bodies, whilst allowing the researcher to justify the use of OS in their research (Levin et al., 2016).

The diversity of OS methods may be intimidating to the researcher but increasing awareness and uptake of such practices will make the process more commonplace, with the potential for OS courses to be undertaken (Toelch & Ostwald, 2018). Altering researcher cultures towards the practice of OS is often noted as the most difficult task when trying to make OS the norm. OS workshops focusing on approaches to OS and why it should be practiced should be made available where applicable to breed an understanding of its importance (Ignat & Ayris, 2021).

What is clear is that the lack of widely implemented OS is frequently not the fault or choice of a researcher. It is more commonly a universal shortcoming, where funding and financial security provide the incentives to fulfil (or not) OS criteria within biodiversity CS and broader scientific research. However, the benefits of OS far outweigh financial savings. Increased collaboration as a guaranteed result of OS is more than likely to provide return of investment at a greater level than initial capital projections may predict. As we are at a crucial point in world history, with mass extinction threatening ecosystem functioning and human survival, it seems that investment in OS should be non-debatable for the success of biodiversity research and the scientific process.

### Guidelines and recommendations for open science in citizen science projects

The results of this study highlight areas where OS practices can be improved in CS projects. It must be noted that OS should not be prescriptive but suggestive, implementing practices where applicable and necessary. As such, OS practices that should be encouraged where applicable include:

1. When creating a citizen science project, a preregister should be made publicly available detailing the aims, methods, analysis, and dissemination of results intended at the start of the project. Where possible, the use of the OSF for publishing should be engaged if the target journal does not offer this service.
2. A data management plan should be created detailing the collection, analysis, and storage of data using online creation tools such as DMPOnline if your organisation does not offer an alternative. These should be available on the project website (if applicable), as supplementary material, or published in the intended journal as well as on DMPOnline.
3. Data and, where relevant, code should be made publicly available as supplementary material or on repositories such as github/GBIF, etc. and linked within the published journal article/on project websites (if applicable). Nondisclosure of sensitive data (where ethics and anonymisation cannot be instigated) should be justified within a data statement.
4. Where possible/applicable, projects should consider the use of open software for replicability for researchers as well as useability for the public.
5. Project results should be published under an open access license and on project websites, freely available to the public.

## Conclusions

CS is considered an OS practice that is implemented most often in biodiversity monitoring. Here, CS can be both a result of OS and an instigator. However, previous studies show that CS projects often do not adhere to OS practices, hindering its potential to reach the goals of OS. The results of this review show that although interest in CS has increased in biodiversity monitoring over time, the openness of such projects has not risen with this. Although principles of OS need improvement, the areas that need addressing specifically appear to be around the use of preregisters and data management plans, which should be implemented at the start of a project. Guidelines are set out to advise projects on how they can initiate more OS principles within CS projects, whilst OS is actively encouraged on a larger scale through instigation within organisations, institutions, and governments allowing scientific research to become more comprehensible, collaborative, and transparent.

## Supplementary Information

Below is the link to the electronic supplementary material.

Online Resource 1 (DOCX 57 KB)

## Data Availability

The datasets generated during and/or analysed during the current study are available in the supplementary materials.
