# Supplementary material for: Do biodiversity monitoring citizen science surveys meet the core principles of open science practices?
Source: Environ Monit Assess. 2023 Jan 12;195(2):295. doi: 10.1007/s10661-022-10887-y (PMC9836331; doi:10.1007/s10661-022-10887-y)
Supplement: Supplementary file 1 — Online Resource 1 (DOCX 57 KB) [file 10661_2022_10887_MOESM1_ESM.docx]

*Supplementary Material*

**Table 1** The 153 studies identified after the methods sorting. The final 42 studies utilised for all analysis are highlighted in green

| Authors | Article Title | Publication Year |
| --- | --- | --- |
| Becker, CD; Agreda, A; Astudillo, E; Costantino, M; Torres, P | Community-based monitoring of fog capture and biodiversity at Loma Alta, Ecuador enhance social capital and institutional cooperation | 2005 |
| Pieterson, E. Corrie; Addison, Lindsay M.; Agobian, Jorge N.; Brooks-Solveson, Brenda; Cassani, John; Everham, Edwin M., III | Five years of the Southwest Florida Frog Monitoring Network: Changes in frog communities as an indicator of landscape change | 2006 |
| Goffredo, Stefano; Pensa, Francesco; Neri, Patrizia; Orlandi, Antonio; Gagliardi, Maria Scola; Velardi, Angela; Piccinetti, Corrado; Zaccanti, Francesco | Unite research with what citizens do for fun: recreational monitoring'' of marine biodiversity | 2010 |
| Stafford, Richard; Hart, Adam G.; Collins, Laura; Kirkhope, Claire L.; Williams, Rachel L.; Rees, Samuel G.; Lloyd, Jane R.; Goodenough, Anne E. | Eu-Social Science: The Role of Internet Social Networks in the Collection of Bee Biodiversity Data | 2010 |
| Suzuki, Takao; Sasaki, Miki | Civil procedure for researching benthic invertebrate animals inhabiting tidal flats in eastern Japan | 2010 |
| Arvanitidis, Christos; Faulwetter, Sarah; Chatzigeorgiou, Georgios; Penev, Lyubomir; Banki, Olaf; Dailianis, Thanos; Pafilis, Evangelos; Kouratoras, Michail; Chatzinikolaou, Eva; Fanini, Lucia; Vasileiadou, Aikaterini; Pavloudi, Christina; Vavilis, Panagiotis; Koulouri, Panayota; Dounas, Costas sara bois | Engaging the broader community in biodiversity research: the concept of the COMBER pilot project for divers in ViBRANT | 2011 |
| Bramanti, Lorenzo; Vielmini, Ilaria; Rossi, Sergio; Stolfa, Stefano; Santangelo, Giovanni | Involvement of recreational scuba divers in emblematic species monitoring: The case of Mediterranean red coral (Corallium rubrum) | 2011 |
| Davies, L.; Bell, J. N. B.; Bone, J.; Head, M.; Hill, L.; Howard, C.; Hobbs, S. J.; Jones, D. T.; Power, S. A.; Rose, N.; Ryder, C.; Seed, L.; Stevens, G.; Toumi, R.; Voulvoulis, N.; White, P. C. L. | Open Air Laboratories (OPAL): A community-driven research programme | 2011 |
| De Angelo, Carlos; Paviolo, Agustin; Rode, Daniela; Cullen, Laury, Jr.; Sana, Denis; Abreu, Kaue Cachuba; da Silva, Marina Xavier; Bertrand, Anne-Sophie; Haag, Taiana; Lima, Fernando; Rinaldi, Alcides Ricieri; Fernandez, Sixto; Ramirez, Fredy; Velazquez, Myriam; Corio, Cristian; Hasson, Esteban; Di Bitetti, Mario S. | Participatory networks for large-scale monitoring of large carnivores: pumas and jaguars of the Upper Parana Atlantic Forest | 2011 |
| Kremen, C.; Ullmann, K. S.; Thorp, R. W. | Evaluating the Quality of Citizen-Scientist Data on Pollinator Communities | 2011 |
| Belt, Jami J.; Krausman, Paul R. | Evaluating Population Estimates of Mountain Goats Based on Citizen Science | 2012 |
| Cox, T. E.; Philippoff, J.; Baumgartner, E.; Smith, C. M. | Expert variability provides perspective on the strengths and weaknesses of citizen-driven intertidal monitoring program | 2012 |
| Deguines, Nicolas; Julliard, Romain; de Flores, Mathieu; Fontaine, Colin | The Whereabouts of Flower Visitors: Contrasting Land-Use Preferences Revealed by a Country-Wide Survey Based on Citizen Science | 2012 |
| Gollan, John; de Bruyn, Lisa Lobry; Reid, Nick; Wilkie, Lance | Can Volunteers Collect Data that are Comparable to Professional Scientists? A Study of Variables Used in Monitoring the Outcomes of Ecosystem Rehabilitation | 2012 |
| Tonachella, Nicolo; Nastasi, Aurora; Kaufman, Gregory; Maldini, Daniela; Rankin, Robert William | Predicting trends in humpback whale (Megaptera novaeangliae) abundance using citizen science | 2012 |
| Slade, Eleanor M.; Merckx, Thomas; Riutta, Terhi; Bebber, Daniel P.; Redhead, David; Riordan, Philip; Macdonald, David W. | Life-history traits and landscape characteristics predict macro-moth responses to forest fragmentation | 2013 |
| Bodilis, P.; Louisy, P.; Draman, M.; Arceo, H. O.; Francour, P. | Can Citizen Science Survey Non-indigenous Fish Species in the Eastern Mediterranean Sea? | 2014 |
| Buesching, Christina D.; Newman, Chris; Macdonald, David W. | How dear are deer volunteers: the efficiency of monitoring deer using teams of volunteers to conduct pellet group counts | 2014 |
| Bulleri, Fabio; Benedetti-Cecchi, Lisandro | Chasing fish and catching data: recreational spearfishing videos as a tool for assessing the structure of fish assemblages on shallow rocky reefs | 2014 |
| Casanovas, Paula; Lynch, Heather J.; Fagan, William F. | Using citizen science to estimate lichen diversity | 2014 |
| Graham, Jason R.; Tan, Qin; Jones, Linda C.; Ellis, James D. | Native Buzz: Citizen scientists creating nesting habitat for solitary bees and wasps | 2014 |
| Weinstein, Anna; Trocki, Linda; Levalley, Ron; Doster, Robert H.; Distler, Trish; Krieger, Katherine | A FIRST POPULATION ASSESSMENT OF BLACK OYSTERCATCHER HAEMATOPUS BACHMANI IN CALIFORNIA | 2014 |
| Arévalo, J.Edgardo; Méndez, Yoryineth; Roberts, Mia; Alvarado, Geiner; Vargas, Sergio | Monitoring species of mammals using track collection by rangers in the Tilarán mountain range, Costa Rica | 2015 |
| Barlow, K. E.; Briggs, P. A.; Haysom, K. A.; Hutson, A. M.; Lechiara, N. L.; Racey, P. A.; Walsh, A. L.; Langton, S. D. | Citizen science reveals trends in bat populations: The National Bat Monitoring Programme in Great Britain | 2015 |
| Biggs, Jeremy; Ewald, Naomi; Valentini, Alice; Gaboriaud, Coline; Dejean, Tony; Griffiths, Richard A.; Foster, Jim; Wilkinson, John W.; Arnell, Andy; Brotherton, Peter; Williams, Penny; Dunn, Francesca | Using eDNA to develop a national citizen science-based monitoring programme for the great crested newt (Triturus cristatus) | 2015 |
| Bosch, Stefan; Lachmann, Lars | Population trends of abundant garden birds in Baden-Wurttemberg 2005-2014: Results of the first 10 years of the citizen-science project 'Hour of the Garden Birds'. | 2015 |
| Branchini, Simone; Pensa, Francesco; Neri, Patrizia; Tonucci, Bianca Maria; Mattielli, Lisa; Collavo, Anna; Sillingardi, Maria Elena; Piccinetti, Corrado; Zaccanti, Francesco; Goffredo, Stefano | Using a citizen science program to monitor coral reef biodiversity through space and time | 2015 |
| Buldrini, Fabrizio; Simoncelli, Antinisca; Accordi, Stefania; Pezzi, Giovanna; Dallai, Daniele | Ten years of citizen science data collection of wetland plants in an urban protected area | 2015 |
| Cartwright, Lyndsay A.; Cvetkovic, Maja; Graham, Spencer; Tozer, Douglas; Chow-Fraser, Patricia | URBAN: Development of a Citizen Science Biomonitoring Program Based in Hamilton, Ontario, Canada | 2015 |
| Hawthorne, T. L.; Elmore, V.; Strong, A.; Bennett-Martin, P.; Finnie, J.; Parkman, J.; Harris, T.; Singh, J.; Edwards, L.; Reed, J. | Mapping non-native invasive species and accessibility in an urban forest: A case study of participatory mapping and citizen science in Atlanta, Georgia | 2015 |
| Kirkendale, L.; Slack-Smith, S.; Fromont, J.; Teufel, D.; Short, P.; Richards, Z.; Hosie, A.; Bryce, M.; Read, S.; Gomez, O. | RESULTS OF THE FIRST INTERTIDAL CITIZEN SCIENCE PROJECT IN WA: PORT HEDLAND COMMUNITY REEF SURVEY MARCH APRIL 2014 | 2015 |
| Wilson, John-James; Jisming-See, Shi-Wei; Brandon-Mong, Guo-Jie; Lim, Aik-Hean; Lim, Voon-Ching; Lee, Ping-Shin; Sing, Kong-Wah | Citizen Science: The First Peninsular Malaysia Butterfly Count | 2015 |
| Abe, Jonathan; Alop-Mabuti, Aleena; Burger, Peyton; Button, Jackson; Ellsberry, Madeline; Hitzeman, Jaycinth; Morgenstern, David; Nunies, Kasey; Strother, Mara; Darling-Munson, Jared; Chan, Yvonne L.; Cassady, Robert; Vasconcellos, Sarah Maile K.; Iseman, Michael D.; Chan, Edward D.; Honda, Jennifer R. | Comparing the temporal colonization and microbial diversity of showerhead biofilms in Hawai'i and Colorado | 2016 |
| Crucitti, Pierangelo; Brocchieri, Davide; Bubbico, Francesco; Tringali, Luca; Vigliotti, Francesco | The employment of Citizen Science the study of biodiversity in a case from the Campagna Romana (Latium) | 2016 |
| Dolrenry, Stephanie; Hazzah, Leela; Frank, Laurence G. | Conservation and monitoring of a persecuted African lion population by Maasai warriors | 2016 |
| Flower, Emily; Jones, Darryl; Bernede, Lilia | Can Citizen Science Assist in Determining Koala (Phascolarctos cinereus) Presence in a Declining Population? | 2016 |
| Gerovasileiou, Vasilis; Dailianis, Thanos; Panteri, Emmanouela; Michalakis, Nikitas; Gatti, Giulia; Sini, Maria; Dimitriadis, Charalampos; Issaris, Yiannis; Salomidi, Maria; Filiopoulou, Irene; Dogan, Alper; d'Avray, Laure Thierry de Ville; David, Romain; Cinar, Melih Ertan; Koutsoubas, Drosos; Feral, Jean-Pierre; Arvanitidis, Christos | CIGESMED for divers: Establishing a citizen science initiative for the mapping and monitoring of coralligenous assemblages in the Mediterranean Sea | 2016 |
| Hardwick, Bess; Kaartinen, Riikka; Koponen, Martti; Roslin, Tomas | A rapid assessment of a poorly known insect group | 2016 |
| Katani, Josiah Z.; Mustalahti, Irmeli; Mukama, Kusaga; Zahabu, Eliakimu | Participatory forest carbon assessment in south-eastern Tanzania: experiences, costs and implications for REDD plus initiatives | 2016 |
| Ladin, Zachary S.; Higgins, Conor D.; Schmit, John Paul; Sanders, Geoffrey; Johnson, Mark J.; Weed, Aaron S.; Marshall, Matthew R.; Campbell, J. Patrick; Comiskey, James A.; Shriver, W. Gregory | Using regional bird community dynamics to evaluate ecological integrity within national parks | 2016 |
| Mahard, Tyler J.; Litvaitis, John A.; Tate, Patrick; Reed, Gregory C.; Broman, Derek J. A. | An Evaluation of Hunter Surveys to Monitor Relative Abundance of Bobcats | 2016 |
| Roelfsema, Chris; Thurstan, Ruth; Beger, Maria; Dudgeon, Christine; Loder, Jennifer; Kovacs, Eva; Gallo, Michele; Flower, Jason; Cabrera, K-le Gomez; Ortiz, Juan; Lea, Alexandra; Kleine, Diana | A Citizen Science Approach: A Detailed Ecological Assessment of Subtropical Reefs at Point Lookout, Australia | 2016 |
| Campanaro, Alessandro; Hardersen, Sonke; De Zan, Lara Redolfi; Antonini, Gloria; Bardiani, Marco; Maura, Michela; Maurizi, Emanuela; Mosconi, Fabio; Zauli, Agnese; Bologna, Marco Alberto; Roversi, Pio Federico; Peverieri, Giuseppino Sabbatini; Mason, Franco | Analyses of occurrence data of protected insect species collected by citizens in Italy | 2017 |
| Davis, Adrian; Major, Richard E.; Taylor, Charlotte E.; Martin, John M. | Novel tracking and reporting methods for studying large birds in urban landscapes | 2017 |
| Ens, E. J.; Bentley-Toon, S.; Campion, F.; Campion, S.; Kelly, J.; Towler, G. | Rapid appraisal links feral buffalo with kunkod (Melaleuca spp.) decline in freshwater billabongs of tropical northern Australia | 2017 |
| Ganey, Joseph L.; Iniguez, Jose M.; Sanderlin, Jamie S.; Block, William M. | Developing a Monitoring Program for Bird Populations in the Chiricahua Mountains, Arizona, Using Citizen Observers: Initial Stages. | 2017 |
| Kallimanis, A. S.; Panitsa, M.; Dimopoulos, P. | Quality of non-expert citizen science data collected for habitat type conservation status assessment in Natura 2000 protected areas | 2017 |
| Kays, Roland; Parsons, Arielle W.; Baker, Megan C.; Kalies, Elizabeth L.; Forrester, Tavis; Costello, Robert; Rota, Christopher T.; Millspaugh, Joshua J.; McShea, William J. | Does hunting or hiking affect wildlife communities in protected areas? | 2017 |
| Long, Seh-Ling; Azmi, Nazirul A. | USING PHOTOGRAPHIC IDENTIFICATION TO MONITOR SEA TURTLE POPULATIONS AT PERHENTIAN ISLANDS MARINE PARK IN MALAYSIA | 2017 |
| Matabos, Marjolaine; Hoeberechts, Maia; Doya, Carol; Aguzzi, Jacopo; Nephin, Jessica; Reimchen, Thomas E.; Leaver, Steve; Marx, Roswitha M.; Albu, Alexandra Branzan; Fier, Ryan; Fernandez-Arcaya, Ulla; Juniper, S. Kim | Expert, Crowd, Students or Algorithm: who holds the key to deep-sea imagery 'big data' processing? | 2017 |
| Mendez, Marcos; de Jaime, Chabier; Alcantara, Manuel A. | Habitat description and interannual variation in abundance and phenology of the endangered beetle Lucanus cervus L. (Coleoptera) using citizen science monitoring | 2017 |
| Newson, Stuart E.; Evans, Hazel E.; Gillings, Simon; Jarrett, David; Raynor, Robert; Wilson, Mark W. | Large-scale citizen science improves assessment of risk posed by wind farms to bats in southern Scotland | 2017 |
| Shupe, Scott M. | High resolution stream water quality assessment in the Vancouver, British Columbia region: a citizen science study | 2017 |
| Suzuki-Ohno, Yukari; Yokoyama, Jun; Nakashizuka, Tohru; Kawata, Masakado | Utilization of photographs taken by citizens for estimating bumblebee distributions | 2017 |
| Austen, Gail E.; Bindemann, Markus; Griffiths, Richard A.; Roberts, David L. | Species identification by conservation practitioners using online images: accuracy and agreement between experts | 2018 |
| Barrows, A. P. W.; Cathey, S. E.; Petersen, C. W. | Marine environment microfiber contamination: Global patterns and the diversity of microparticle origins | 2018 |
| Campbell, Heather; Engelbrecht, Ian | The Baboon Spider Atlas - using citizen science and the fear factor' to map baboon spider (Araneae: Theraphosidae) diversity and distributions in Southern Africa | 2018 |
| Eritja, Roger; Rubido-Bara, Marga; Delacour-Estrella, Sarah; Bengoa, Mikel; Ruiz-Arrondo, Ignacio | Citizen science and biodiversity: first record of Aedes (Fredwardsius) vittatus (Bigot, 1861) (Diptera, Culicidae) in Galicia, by the means of the Mosquito Alert platform | 2018 |
| Farhadinia, Mohammad S.; Moll, Remington J.; Montgomery, Robert A.; Ashrafi, Sohrab; Johnson, Paul J.; Hunter, Luke T. B.; Macdonald, David W. | Citizen science data facilitate monitoring of rare large carnivores in remote montane landscapes | 2018 |
| Jones, Fiona M.; Allen, Campbell; Arteta, Carlos; Arthur, Joan; Black, Caitlin; Emmerson, Louise M.; Freeman, Robin; Hines, Greg; Lintott, Chris J.; Machackova, Zuzana; Miller, Grant; Simpson, Rob; Southwell, Colin; Torsey, Holly R.; Zisserman, Andrew; Hart, Tom | Time-lapse imagery and volunteer classifications from the Zooniverse Penguin Watch project | 2018 |
| Killion, Alexander K.; Roloff, Gary J.; Mayhew, Sarah; Campa, Henry, III; Winterstein, Scott | Implementing and Evaluating a Citizen-Science Program to Support Wildlife Management: MI-MAST | 2018 |
| Kortmann, Mareike; Heurich, Marco; Latifi, Hooman; Roesner, Sascha; Seidl, Rupert; Mueller, Joeg; Thorn, Simon | Forest structure following natural disturbances and early succession provides habitat for two avian flagship species, capercaillie (Tetrao urogallus) and hazel grouse (Tetrastes bonasia) | 2018 |
| Marizzi, Christine; Florio, Antonia; Lee, Melissa; Khalfan, Mohammed; Ghiban, Cornel; Nash, Bruce; Dorey, Jenna; McKenzie, Sean; Mazza, Christine; Cellini, Fabiana; Baria, Carlo; Bepat, Ron; Cosentino, Lena; Dvorak, Alexander; Gacevic, Amina; Guzman-Moumtzis, Cristina; Heller, Francesca; Holt, Nicholas Alexander; Horenstein, Jeffrey; Joralemon, Vincent; Kaur, Manveer; Kaur, Tanveer; Khan, Armani; Kuppan, Jessica; Laverty, Scott; Lock, Camila; Pena, Marianne; Petrychyn, Ilona; Puthenkalam, Indu; Ram, Daval; Ramos, Arlene; Scoca, Noelle; Sin, Rachel; Gonzalez, Izabel; Thakur, Akansha; Usmanov, Husan; Han, Karen; Wu, Andy; Zhu, Tiger; Micklos, David Andrew | DNA barcoding Brooklyn (New York): A first assessment of biodiversity in Marine Park by citizen scientists | 2018 |
| Martay, B.; Pearce-Higgins, J. W. | Using data from schools to model variation in soil invertebrates across the UK: The importance of weather, climate, season and habitat | 2018 |
| Martay, Blaise; Pearce-Higgins, James W.; Harris, Sarah J.; Gillings, Simon | Monitoring landscape-scale environmental changes with citizen scientists: Twenty years of land use change in Great Britain | 2018 |
| Micaroni, Valerio; Strano, Francesca; Di Franco, Davide; Langeneck, Joachim; Gravili, Cinzia; Bertolino, Marco; Costa, Gabriele; Rindi, Fabio; Froglia, Carlo; Crocetta, Fabio; Giangrande, Adriana; Nicoletti, Luisa; Medagli, Pietro; Zuccarello, Vincenzo; Arzeni, Stefano; Bo, Marzia; Betti, Federico; Mastrototaro, Francesco; Lattanzi, Loretta; Piraino, Stefano; Boero, Ferdinando | Project Biodiversity MARE Tricase: biodiversity research, monitoring and promotion at MARE Outpost (Apulia, Italy) | 2018 |
| Rykken, Jessica J.; Farrell, Brian D. | Exploring the Microwilderness of Boston Harbor Islands National Recreation Area: Terrestrial Invertebrate All Taxa Biodiversity Inventory | 2018 |
| Singh, Priyanka; Saran, Sameer; Kumar, Dheeraj; Padalia, Hitendra; Srivastava, Ashutosh; Kumar, A. Senthil | Species Mapping Using Citizen Science Approach Through IBIN Portal: Use Case in Foothills of Himalaya | 2018 |
| Thornhill, Ian; Chautard, Alice; Loiselle, Steven | Monitoring Biological and Chemical Trends in Temperate Still Waters Using Citizen Science | 2018 |
| Baker, D. J.; Clarke, R. H.; McGeoch, M. A. | The power to detect regional declines in common bird populations using continental monitoring data | 2019 |
| Begona Garcia, Maria; Luis Silva, Jose; Tejero, Pablo; Pardo, Iker; Gomez, Daniel | Tracking the long-term dynamics of plant diversity in Northeast Spain with a network of volunteers and rangers | 2019 |
| Beirne, Christopher; Meier, Amelia C.; Mbele, Alex Ebang; Menie, Guillaume Menie; Froese, Graden; Okouyi, Joseph; Poulsen, John R. | Participatory monitoring reveals village-centered gradients of mammalian defaunation in central Africa | 2019 |
| Corazza, Carla; Baraldi, Nicola; Aldrovandi, Stefano; Mazzotti, Stefano | Biodiversity for everyone: the citizen science projects of the Museum of Natural History of Ferrara between research and collections. | 2019 |
| de Juana, Fernando; Monasterio, Yeray; Escobes, Ruth; Luis Albala, Jose; Belamendia, Gorka; de Olano, Ibon; Sebastian, Jose; Webster, Brian | The Macroheterocera (Lepidoptera) of the Salburua wetlands (Vitoria-Gasteiz, Araba/Alava, Spain): a citizen science project | 2019 |
| Drummond, Faline M.; Armstrong, Doug P. | Use of distance sampling to measure long-term changes in bird densities in a fenced wildlife sanctuary | 2019 |
| Franca, Juliana Silva; Solar, Ricardo; Hughes, Robert M.; Callisto, Marcos | Student monitoring of the ecological quality of neotropical urban streams | 2019 |
| Giovos, Ioannis; Kleitou, Periklis; Poursanidis, Dimitris; Batjakas, Ioannis; Bernardi, Giacomo; Crocetta, Fabio; Doumpas, Nikolaos; Kalogirou, Stefanos; Kampouris, Thodoros E.; Keramidas, Ioannis; Langeneck, Joachim; Maximiadi, Mary; Mitsou, Eleni; Stoilas, Vasileios-Orestis; Tiralongo, Francesco; Romanidis-Kyriakidis, Georgios; Xentidis, Nicholas-Jason; Zenetos, Argyro; Katsanevakis, Stelios | Citizen-science for monitoring marine invasions and stimulating public engagement: a case project from the eastern Mediterranean | 2019 |
| Giovos, Ioannis; Stoilas, Vasilis-Orestis; Al-Mabruk, Sara A. A.; Doumpas, Nikolaos; Marakis, Philippos; Maximiadi, Mary; Moutopoulos, Dimitrios; Kleitou, Periklis; Keramidas, Ioannis; Tiralongo, Francesco; de Maddalena, Alessandro | Integrating local ecological knowledge, citizen science and long-term historical data for endangered species conservation: Additional records of angel sharks (Chondrichthyes: Squatinidae) in the Mediterranean Sea | 2019 |
| He, Yurong; Parrish, Julia K.; Rowe, Shawn; Jones, Timothy | Evolving interest and sense of self in an environmental citizen science program | 2019 |
| Hisasue, Y.; Hisamatsu, S.; Murakami, H. | Exotic ant species found by monitoring surveys for suspected red imported fire ants, including submissions by citizens to the Biodiversity Center, Ehime Prefectural Institute of Public Health and Environmental Science during 2017. | 2019 |
| Mason, Lisa; Arathi, H. S. | Assessing the efficacy of citizen scientists monitoring native bees in urban areas | 2019 |
| Matear, Liam; Robbins, James R.; Hale, Michelle; Potts, Jonathan | Cetacean biodiversity in the Bay of Biscay: Suggestions for environmental protection derived from citizen science data | 2019 |
| Perez-Belmont, Patricia; Alvarado, Jannice; Vazquez-Salvador, Nallely; Rodriguez, Erika; Valiente, Elsa; Diaz, Julio | Water quality monitoring in the Xochimilco peri-urban wetland: experiences engaging in citizen science | 2019 |
| Pescott, Oliver L.; Walker, Kevin J.; Harris, Felicity; New, Hayley; Cheffings, Christine M.; Newton, Niki; Jitlal, Mark; Redhead, John; Smart, Simon M.; Roy, David B. | The design, launch and assessment of a new volunteer-based plant monitoring scheme for the United Kingdom | 2019 |
| Puan, Chong Leong; Yeong, Kok Loong; Ong, Kang Woei; Fauzi, Muhd Izzat Ahmad; Yahya, Muhammad Syafiq; Khoo, Swee Seng | Influence of landscape matrix on urban bird abundance: evidence from Malaysian citizen science data | 2019 |
| Qian, Haiyuan; Yu, Jianping; Shen, Xiaoli; Ding, Ping; Li, Sheng | Diversity and composition of birds in the Qianjiangyuan National Park pilot | 2019 |
| Rafiq, Kasim; Bryce, Caleb M.; Rich, Lindsey N.; Coco, Carli; Miller, David A. W.; Meloro, Carlo; Wich, Serge A.; McNutt, John W.; Hayward, Matthew W. | Tourist photographs as a scalable framework for wildlife monitoring in protected areas | 2019 |
| Robinne, Francois-Nicolas; Gallagher, Louise; Brethaut, Christian; Schlaepfer, Martin A. | A novel tool for measuring the penetration of the ecosystem service concept into public policy | 2019 |
| Schade, Sven; Kotsev, Alexander; Cardoso, Ana Cristina; Tsiamis, Konstantinos; Gervasini, Eugenio; Spinelli, Fabiano; Mitton, Irena; Sgnaolin, Roberto | Aliens in Europe. An open approach to involve more people in invasive species detection | 2019 |
| Schuttler, Stephanie G.; Sears, Rebecca S.; Orendain, Isabel; Khot, Rahul; Rubenstein, Daniel; Rubenstein, Nancy; Dunn, Robert R.; Baird, Elizabeth; Kandros, Kimberly; O'Brien, Timothy; Kays, Roland | Citizen Science in Schools: Students Collect Valuable Mammal Data for Science, Conservation, and Community Engagement | 2019 |
| Smale, Dan A.; Epstein, Graham; Parry, Mark; Attrill, Martin J. | Spatiotemporal variability in the structure of seagrass meadows and associated macrofaunal assemblages in southwest England (UK): Using citizen science to benchmark ecological pattern | 2019 |
| Sumner, Seirian; Bevan, Peggy; Hart, Adam G.; Isaac, Nicholas J. B. | Mapping species distributions in 2 weeks using citizen science | 2019 |
| Yardi, Kranti D.; Bharucha, Erach; Girade, Swapnil | Post-restoration monitoring of water quality and avifaunal diversity of Pashan Lake, Pune, India using a citizen science approach | 2019 |
| Appenfeller, Logan R.; Lloyd, Sarah; Szendrei, Zsofia | Citizen science improves our understanding of the impact of soil management on wild pollinator abundance in agroecosystems | 2020 |
| Blake, Charlie; Rhanor, Allison K. | The impact of channelization on macroinvertebrate bioindicators in small order Illinois streams: insights from long-term citizen science research | 2020 |
| Bonnet-Lebrun, A-S; Karamanlidis, A. A.; de Gabriel Hernando, M.; Renner, I; Gimenez, O. | Identifying priority conservation areas for a recovering brown bear population in Greece using citizen science data | 2020 |
| Castracani, Cristina; Spotti, Fiorenza Augusta; Schifani, Enrico; Giannetti, Daniele; Ghizzoni, Martina; Grasso, Donato Antonio; Mori, Alessandra | Public Engagement Provides First Insights on Po Plain Ant Communities and Reveals the Ubiquity of the Cryptic Species Tetramorium immigrans (Hymenoptera, Formicidae) | 2020 |
| Deguines, Nicolas; Prince, Karine; Prevot, Anne-Caroline; Fontaine, Benoit | Assessing the emergence of pro-biodiversity practices in citizen scientists of a backyard butterfly survey | 2020 |
| Ebihaha, Kengo; Yasukawa, Masaki; Nagai, Mihoko; Kitsuregawa, Masaru; Washitani, Izumi | Feasibility of citizen science monitoring of mutualistic networks between butterflies and plants in Tokyo, Japan. | 2020 |
| Edgar, Graham J.; Cooper, Antonia; Baker, Susan C.; Barker, William; Barrett, Neville S.; Becerro, Mikel A.; Bates, Amanda E.; Brock, Danny; Ceccarelli, Daniela M.; Clausius, Ella; Davey, Marlene; Davis, Tom R.; Day, Paul B.; Green, Andrew; Griffiths, Samuel R.; Hicks, Jamie; Hinojosa, Ivan A.; Jones, Ben K.; Kininmonth, Stuart; Larkin, Meryl F.; Lazzari, Natali; Lefcheck, Jonathan S.; Ling, Scott D.; Mooney, Peter; Oh, Elizabeth; Perez-Matus, Alejandro; Pocklington, Jacqueline B.; Riera, Rodrigo; Sanabria-Fernandez, Jose A.; Seroussi, Yanir; Shaw, Ian; Shields, Derek; Shields, Joe; Smith, Margo; Soler, German A.; Stuart-Smith, Jemina; Turnbull, John; Stuart-Smith, Rick D. | Reef Life Survey: Establishing the ecological basis for conservation of shallow marine life | 2020 |
| Gardiner, Tim; Didham, Raphael K. | Glowing, glowing, gone? Monitoring long-term trends in glow-worm numbers in south-east England | 2020 |
| Gili, Fabrizio; Newson, Stuart E.; Gillings, Simon; Chamberlain, Dan E.; Border, Jennifier A. | Bats in urbanising landscapes: habitat selection and recommendations for a sustainable future | 2020 |
| Gizzi, Francesca; Jimenez, Jesus; Schaefer, Susanne; Castro, Nuno; Costa, Sonia; Lourenco, Silvia; Jose, Ricardo; Canning-Clode, Joao; Monteiro, Joao | Before and after a disease outbreak: Tracking a keystone species recovery from a mass mortality event | 2020 |
| Lanner, Julia; Huchler, Katharina; Pachinger, Baerbel; Sedivy, Claudio; Meimberg, Harald | Dispersal patterns of an introduced wild bee, Megachile sculpturalis Smith, 1853 (Hymenoptera: Megachilidae) in European alpine countries | 2020 |
| Monterastelli, Elisa; Poloni, Riccardo | The Insetti.A.MO project: an insect population census in Modena city (Italy) | 2020 |
| Nunes, Miguel Simoes; Falconer, Kristie; Jelic, Dusan; Martin, Thomas Edward; Kucinic, Mladen; Jocque, Merlijn | The value of eco-volunteer projects for biodiversity conservation: butterfly monitoring in Krka National Park (Croatia) with an updated checklist | 2020 |
| Platenberg, Renata J.; Raymore, Martha; Primack, Avram; Troutman, Kelcie | Monitoring Vocalizing Species by Engaging Community Volunteers Using Cell Phones | 2020 |
| Rameli, Nurul I. A. Mohd; Lappan, Susan; Bartlett, Thad Q.; Ahmad, Siti K.; Ruppert, Nadine | Are social media reports useful for assessing small ape occurrence? A pilot study from Peninsular Malaysia | 2020 |
| Schneiderhan-Opel, Jennifer; Bogner, Franz X. | How fascination for biology is associated with students' learning in a biodiversity citizen science project | 2020 |
| Shah, Md Nur Ahad; Khan, Md Kawsar | OdoBD: An online database for the dragonflies and damselflies of Bangladesh | 2020 |
| Shang Xiaotong; Luo Chunping; Li Bin; Zheng Yong; Zhou Zhiqiang; Zhang Li; Li Sheng | Diversity and Fauna Composition of Birds in the Wanglang National Nature Reserve, Sichuan. | 2020 |
| Sheard, Julie K.; Sanders, Nathan J.; Gundlach, Carsten; Schar, Sami; Larsen, Rasmus Stenbak | Monitoring the influx of new species through citizen science: the first introduced ant in Denmark | 2020 |
| Stenhouse, Alan; Roetman, Philip; Lewis, Megan; Koh, Lian Pin | Koala Counter: Recording Citizen Scientists' search paths to Improve Data Quality | 2020 |
| Tiralongo, Francesco; Crocetta, Fabio; Riginella, Emilio; Lillo, Antonio Oscar; Tondo, Elena; Macali, Armando; Mancini, Emanuele; Russo, Fabio; Coco, Salvatore; Paolillo, Giuseppe; Azzurro, Ernesto | Snapshot of rare, exotic and overlooked fish species in the Italian seas: A citizen science survey | 2020 |
| Uhrin, Amy V.; Lippiatt, Sherry; Herring, Carlie E.; Dettloff, Kyle; Bimrose, Kate; Butler-Minor, Chris | Temporal Trends and Potential Drivers of Stranded Marine Debris on Beaches Within Two US National Marine Sanctuaries Using Citizen Science Data | 2020 |
| Werenkraut, Victoria; Baudino, Florencia; Roy, Helen E. | Citizen science reveals the distribution of the invasive harlequin ladybird (Harmonia axyridisPallas) in Argentina | 2020 |
| Wotton, S. R.; Eaton, M. A.; Sheehan, D.; Munyekenye, F. Barasa; Burfield, I. J.; Butchart, S. H. M.; Moleofi, K.; Nalwanga-Wabwire, D.; Ndang'ang'a, P. K.; Pomeroy, D.; Senyatso, K. J.; Gregory, R. D. | Developing biodiversity indicators for African birds | 2020 |
| Ahmad, Abrar; Gary, Demi; Rodiansyah; Sinta; Srifitria; Putra, Wahyu; Sagita, Novia; Adirahmanta, Sadtata Noor; Miller, Adam E. | Leveraging local knowledge to estimate wildlife densities in bornean tropical rainforests | 2021 |
| Alther, Roman; Bongni, Nicole; Borko, Spela; Fiser, Cene; Altermatt, Florian | Citizen science approach reveals groundwater fauna in Switzerland and a new species of Niphargus (Amphipoda, Niphargidae) | 2021 |
| Anton, Victor; Germishuys, Jannes; Bergstrom, Per; Lindegarth, Mats; Obst, Matthias | An open-source, citizen science and machine learning approach to analyse subsea movies | 2021 |
| Arbelaez-Cortes, Enrique; Sanchez-Sarria, Camilo E.; Ocampo, David; Estela, Felipe A.; Garcia-Arroyo, Michelle; MacGregor-Fors, Ian | EXPERIENCES OF SURVEYING URBAN BIRDS DURING THE ANTHROPAUSE IN COLOMBIA | 2021 |
| Aura, Christopher Mulanda; Nyamweya, Chrisphine S.; Owiti, Horace; Odoli, Cyprian; Musa, Safina; Njiru, James M.; Nyakeya, Kobingi; Masese, Frank O. | Citizen Science for Bio-indication: Development of a Community-Based Index of Ecosystem Integrity for Assessing the Status of Afrotropical Riverine Ecosystems | 2021 |
| Balciauskas, Linas; Balciauskiene, Laima; Litvaitis, John A.; Tijusas, Eugenijus | Adaptive monitoring: using citizen scientists to track wolf populations when winter-track counts become unreliable | 2021 |
| Biddle, Rebecca; Solis-Ponce, Ivette; Jones, Martin; Marsden, Stuart; Pilgrim, Mark; Devenish, Christian | The value of local community knowledge in species distribution modelling for a threatened Neotropical parrot | 2021 |
| Encarnacao, Joao; Baptista, Vania; Teodosio, Maria Alexandra; Morais, Pedro | Low-Cost Citizen Science Effectively Monitors the Rapid Expansion of a Marine Invasive Species | 2021 |
| Flaminio, Simone; Ranalli, Rosa; Zavatta, Laura; Galloni, Marta; Bortolotti, Laura | Beewatching: A Project for Monitoring Bees through Photos | 2021 |
| Gadsden, Gabriel, I; Malhotra, Rumaan; Schell, Justin; Carey, Tiffany; Harris, Nyeema C. | Michigan ZoomIN: Validating Crowd-Sourcing to Identify Mammals from Camera Surveys | 2021 |
| Garcia, Maria B.; Silva, Jose L.; Tejero, Pablo; Pardo, Iker | Detecting early-warning signals of concern in plant populations with a Citizen Science network. Are threatened and other priority species for conservation performing worse? | 2021 |
| Gutierrez-Munoz, Paula; Walters, Alice E. M.; Dolman, Sarah J.; Pierce, Graham J. | Patterns and Trends in Cetacean Occurrence Revealed by Shorewatch, a Land-Based Citizen Science Program in Scotland (United Kingdom) | 2021 |
| Kalaentzis, Konstantinos; Kazilas, Christos; Demetriou, Jakovos; Koutsoukos, Evangelos; Avtzis, Dimitrios N.; Georgiadis, Christos | Alientoma, a Dynamic Database for Alien Insects in Greece and Its Use by Citizen Scientists in Mapping Alien Species | 2021 |
| Kasten, Paula; Jenkins, Stuart R.; Christofoletti, Ronaldo A. | Participatory Monitoring-A Citizen Science Approach for Coastal Environments | 2021 |
| Kirchhoff, Casey; Callaghan, Corey T.; Keith, David A.; Indiarto, Dony; Taseski, Guy; Ooi, Mark Kj; Le Breton, Tom D.; Mesaglio, Thomas; Kingsford, Richard T.; Cornwell, William K. | Rapidly mapping fire effects on biodiversity at a large-scale using citizen science | 2021 |
| Lee, Tracy S.; Kahal, Nicole L.; Kinas, Holly L.; Randall, Lea A.; Baker, Tyne M.; Carney, Vanessa A.; Kendell, Kris; Sanderson, Ken; Duke, Danah | Advancing Amphibian Conservation through Citizen Science in Urban Municipalities | 2021 |
| Lin, Meixi; Simons, Ariel Levi; Harrigan, Ryan J.; Curd, Emily E.; Schneider, Fabian D.; Ruiz-Ramos, Dannise V.; Gold, Zack; Osborne, Melisa G.; Shirazi, Sabrina; Schweizer, Teia M.; Moore, Tiara N.; Fox, Emma A.; Turba, Rachel; Garcia-Vedrenne, Ana E.; Helman, Sarah K.; Rutledge, Kelsi; Mejia, Maura Palacios; Marwayana, Onny; Munguia Ramos, Miroslava N.; Wetzer, Regina; Pentcheff, N. Dean; McTavish, Emily Jane; Dawson, Michael N.; Shapiro, Beth; Wayne, Robert K.; Meyer, Rachel S. | Landscape analyses using eDNA metabarcoding and Earth observation predict community biodiversity in California | 2021 |
| Machado, Augusto A.; Bertoncini, Athila A.; Santos, Luciano N.; Creed, Joel C.; Masi, Bruno P. | Participatory monitoring of marine biological invaders: a novel program to include citizen scientists | 2021 |
| Mangelli, Tarcio S.; Zapelini, Cleverson; da Rocha, Wesley Duarte; Schiavetti, Alexandre | Voluntary scuba diving as a method for monitoring invasive exotic marine species | 2021 |
| Mesaglio, Thomas; Soh, Aaron; Kurniawidjaja, Steven; Sexton, Chuck | 'First Known Photographs of Living Specimens': the power of iNaturalist for recording rare tropical butterflies | 2021 |
| Meschini, Marta; Machado Toffolo, Mariana; Marchini, Chiara; Caroselli, Erik; Prada, Fiorella; Mancuso, Arianna; Franzellitti, Silvia; Locci, Laura; Davoli, Marco; Trittoni, Michele; Nanetti, Enrico; Tittarelli, Mara; Bentivogli, Riccardo; Branchini, Simone; Neri, Patrizia; Goffredo, Stefano | Reliability of Data Collected by Volunteers: A Nine-Year Citizen Science Study in the Red Sea | 2021 |
| Meyer, Rachel S.; Ramos, Miroslava Munguia; Lin, Meixi; Schweizer, Teia M.; Gold, Zachary; Ramos, Dannise Ruiz; Shirazi, Sabrina; Kandlikar, Gaurav; Kwan, Wai-Yin; Curd, Emily E.; Freise, Amanda; Parker, Jordan Moberg; Sexton, Jason P.; Wetzer, Regina; Pentcheff, N. Dean; Wall, Adam R.; Pipes, Lenore; Garcia-Vedrenne, Ana; Mejia, Maura Palacios; Moore, Tiara; Orland, Chloe; Ballare, Kimberly M.; Worth, Anna; Beraut, Eric; Aronson, Emma L.; Nielsen, Rasmus; Lewin, Harris A.; Barber, Paul H.; Wall, Jeff; Kraft, Nathan; Shapiro, Beth; Wayne, Robert K. | The CALeDNA program: Citizen scientists and researchers inventory California's biodiversity | 2021 |
| Moller, Anders Pape; Czeszczewik, Dorota; Erritzoe, Johannes; Flensted-Jensen, Einar; Laursen, Karsten; Liang, Wei; Walankiewicz, Wieslaw | Citizen Science for Quantification of Insect Abundance on Windshields of Cars Across Two Continents | 2021 |
| Moro, Arrigo; Beaurepaire, Alexis; Dall'Olio, Raffaele; Rogenstein, Steve; Blacquiere, Tjeerd; Dahle, Bjorn; de Miranda, Joachim R.; Dietemann, Vincent; Locke, Barbara; Licon Luna, Rosa Maria; Le Conte, Yves; Neumann, Peter | Using Citizen Science to Scout Honey Bee Colonies That Naturally Survive Varroa destructor Infestations | 2021 |
| Pellicioli, Luca; Cimberio, Patrizia | Citizen science project on Alpine ibex, Capra ibex, in the Orobie Alps. | 2021 |
| Rodhouse, Thomas J.; Rose, Sara; Hawkins, Trent; Rodriguez, Rogelio M. | Audible bats provide opportunities for citizen scientists | 2021 |
| Rowe, Helen, I; Gruber, Daniel; Fastiggi, Mary | Where to start? A new citizen science, remote sensing approach to map recreational disturbance and other degraded areas for restoration planning | 2021 |
| Sanden, Taru; Wawra, Anna; Berthold, Helene; Miloczki, Julia; Schweinzer, Agnes; Gschmeidler, Brigitte; Spiegel, Heide; Debeljak, Marko; Trajanov, Aneta | TeaTime4Schools: Using Data Mining Techniques to Model Litter Decomposition in Austrian Urban School Soils | 2021 |
| Squires, Thomas M.; Yuda, Pramana; Akbar, Panji Gusti; Collar, Nigel J.; Devenish, Christian; Taufiqurrahman, Imam; Wibowo, Waskito Kukuh; Winarni, Nurul L.; Yanuar, Ahmad; Marsden, Stuart J. | Citizen science rapidly delivers extensive distribution data for birds in a key tropical biodiversity area | 2021 |
| Stenhouse, Alan; Perry, Tahlia; Grutzner, Frank; Lewis, Megan; Koh, Lian Pin | EchidnaCSI - Improving monitoring of a cryptic species at continental scale using Citizen Science | 2021 |
| Sun, Catherine C.; Hurst, Jeremy E.; Fuller, Angela K. | Citizen Science Data Collection for Integrated Wildlife Population Analyses | 2021 |
| Thomaes, Arno; Barbalat, Sylvie; Bardiani, Marco; Bower, Laura; Campanaro, Alessandro; Fanega Sleziak, Natalia; Goncalo Soutinho, Joao; Govaert, Sanne; Harvey, Deborah; Hawes, Colin; Kadej, Marcin; Mendez, Marcos; Meriguet, Bruno; Rink, Markus; Rossi De Gasperis, Sarah; Ruyts, Sanne; Jelaska, Lucija Seric; Smit, John; Smolis, Adrian; Snegin, Eduard; Tagliani, Arianna; Vrezec, Al | The European Stag Beetle (Lucanus cervus) Monitoring Network: International Citizen Science Cooperation Reveals Regional Differences in Phenology and Temperature Response | 2021 |
| Townsend, Philip A.; Clare, John D. J.; Liu, Nanfeng; Stenglein, Jennifer L.; Anhalt-Depies, Christine; Van Deelen, Timothy R.; Gilbert, Neil A.; Singh, Aditya; Martin, Karl J.; Zuckerberg, Benjamin webster | Snapshot Wisconsin: networking community scientists and remote sensing to improve ecological monitoring and management | 2021 |
| Yang, Jun; Xing, Danqi; Luo, Xiangyu | Assessing the performance of a citizen science project for monitoring urban woody plant species diversity in China | 2021 |

**Table 2** RAW data for openness scores per principle: -1 (closed), 0.5 (partially open), 1 (open). Blanks are found where the principle was not applicable (NA).

| Openness Scores Per Principle | | | | | |
| --- | --- | --- | --- | --- | --- |
| DMP | Preregistration | Data | Code | Software | Access |
| 0.5 | 1 | 1 |  | 1 | -1 |
| -1 | -1 | 1 | 1 |  | 1 |
| -1 | -1 | -1 | -1 | 1 | -1 |
| -1 | 0.5 | 1 | 0.5 | -1 | -1 |
| -1 | -1 | 1 | -1 | -1 | -1 |
| -1 | -1 | -1 | -1 | 1 | -1 |
| 1 | -1 | 1 |  |  | 1 |
| -1 | 0.5 | 0.5 |  | 0.5 | 1 |
| 1 | -1 | 1 |  |  | 1 |
| 0.5 | 1 | 0.5 |  |  | 1 |
| 1 |  | 1 | 0.5 | 0.5 | 1 |
| -1 | -1 | 0.5 |  | -1 | 1 |
| 0.5 | -1 | 1 | -1 | 1 | 1 |
| 0.5 | 0.5 | 0.5 |  |  | 1 |
| 0.5 | -1 | 1 | 1 | 1 | 1 |
| -1 | -1 | 0.5 |  |  | -1 |
| -1 | 0.5 | 1 |  | 0.5 | 1 |
| 0.5 | -1 | 1 | 1 | 1 | 1 |
| 0.5 | -1 | 0.5 | 0.5 | 1 | 1 |
|  | 0.5 | 1 | 1 | 1 | 1 |
| -1 | 1 | 1 | 1 | 0.5 | -1 |
| 0.5 | -1 | 1 | 1 | 1 | 1 |
| -1 | -1 | 1 | 0.5 | 1 | -1 |
| 0.5 | 0.5 | 1 |  | -1 | 1 |
| -1 | -1 | 1 | 1 | 1 | -1 |
| -1 | -1 | 0.5 | 0.5 | 1 | -1 |
| -1 | 0.5 | 1 |  |  | -1 |
| -1 | -1 | 1 |  | 1 | 1 |
| 1 | 0.5 | 1 |  |  | 1 |
| -1 | -1 | 1 | 1 | 0.5 | 1 |
| 0.5 | -1 | 1 | -1 | 1 | 1 |
| -1 | 0.5 | -1 | -1 | 0.5 | 1 |
| 1 | -1 | 1 | 1 | 1 | 1 |
| -1 | -1 | -1 | -1 | -1 | -1 |
| -1 | -1 | 0.5 | 0.5 | 0.5 | 1 |
| -1 | -1 | 1 | -1 | 1 | 1 |
| -1 | -1 | 1 |  |  | -1 |
| -1 | 0.5 | 1 |  | 0.5 | 1 |
| -1 | -1 | -1 |  | 0.5 | -1 |
| -1 | 0.5 | 1 | 0.5 | 1 | 1 |
| -1 | -1 | 1 | 0.5 | 1 | 1 |
| -1 | -1 | 1 | -1 | 1 | -1 |

**Table 3** Data for the number of citizen science projects per year and associated average annual openness score.

| Year | Number of Projects | Average Annual Open Score |
| --- | --- | --- |
| 2005 | 1 | 0.5 |
| 2010 | 1 | 0.2 |
| 2011 | 1 | -0.67 |
| 2012 | 1 | -0.17 |
| 2015 | 3 | -0.39 |
| 2016 | 4 | 0.55 |
| 2017 | 6 | 0.18 |
| 2018 | 2 | 0.5 |
| 2019 | 4 | 0.35 |
| 2020 | 8 | 0.22 |
| 2021 | 11 | -0.05 |
